# Induction of Premalignant Host Responses by Cathepsin X/Z-Deficiency in *Helicobacter Pylori-*Infected Mice

**DOI:** 10.1371/journal.pone.0070242

**Published:** 2013-07-30

**Authors:** Sabine Krueger, Anja Bernhardt, Thomas Kalinski, Martin Baldensperger, Michael Zeh, Anne Teller, Daniela Adolf, Thomas Reinheckel, Albert Roessner, Doerthe Kuester

**Affiliations:** 1 Department of Pathology, Medical Informatics, Otto-von-Guericke University, Magdeburg, Germany; 2 Department of Biometry and Medical Informatics, Otto-von-Guericke University, Magdeburg, Germany; 3 Institute of Molecular Medicine and Cell Research and BIOSS Centre for Biological Signalling Studies, Albert-Ludwigs-University, Freiburg, Germany; Institut Pasteur Paris, France

## Abstract

*Helicobacter pylori* are responsible for the induction of chronic gastric inflammation progressing to atrophy, metaplasia, and gastric cancer. The overexpression of Cathepsin X/Z (Ctsz) in *H. pylori-*infected mucosa and gastric cancer is mediated predominantly by an augmented migration of ctsz^−/−^positive macrophages and the up-regulation of Ctsz in tumor epithelium. To explore the Ctsz-function in the context of chronic inflammation and the development of preneoplastic lesions, we used *Ctsz*-deficient mice in a *H. pylori* gastritis model. *Ctsz* ^−/−^ and wild-type (wt) mice were infected with *H. pylori* strain SS1. The mice were sacrificed at 24, 36, and 50 weeks post infection (wpi). The stomach was removed, and gastric strips were snap-frozen or embedded and stained with H&E. Tissue sections were scored for epithelial lesions and inflammation. Ki-67 and F4/80 immunostaining were used to measure epithelial cell proliferation and macrophage infiltration, respectively. The upregulation of compensating cathepsins and cytokines were confirmed by Western blotting and quantitative RT-PCR. SS1-infected wt and *ctsz* ^−/−^ mice showed strong inflammation, foveolar hyperplasia, atrophy, and cystically-dilated glands. However, at 50 wpi, *ctsz* ^−/−^ mice developed significantly more severe spasmolytic polypeptide-expressing metaplasia (SPEM), showed enhanced epithelial proliferation, and higher levels of infiltrating macrophages. Induction of cytokines was higher and significantly prolonged in *ctsz* ^−/−^ mice compared to wt. Ctsz deficiency supports *H. pylori*-dependent development of chronic gastritis up to metaplasia, indicating a protective, but not proteolytic, function of Ctsz in inflammatory gastric disease.

## Introduction

Persistent *H. pylori* infection is one major cause of gastric cancer. The *H. pylori*-dependent activation of diverse signaling cascades induces the upregulation of proinflammatory chemokines and induces morphological rearrangements of epithelial cells, resulting in chronic gastritis, which progresses from atrophy, to intestinal and spasmolytic metaplasia, dysplasia, and finally to cancer. This diverse clinical outcome may be associated with the expression of bacterial virulence factors. Two major virulence factors have been studied extensively, the cytotoxin-associated antigen A (CagA) and the vacuolating cytotoxin A (VacA) [1].

Proteolysis is instrumental for extracellular matrix (ECM) degradation during tumor invasion and metastasis. However, the proteases involved are not solely produced by cancer cells. The activated tumor microenvironment, including inflammatory immune cells, such as macrophages, supplies many active proteases already during premalignant stages of tumorigenesis [2], [3]. *H. pylori* has been described in conjunction with increased expression of certain matrix metalloproteinases (MMPs), such as MMP-1, MMP-7, or MMP-9 [4], [5], [6], [7], [8], [9]. In contrast, among the cysteine proteases, only one cathepsin was found to be upregulated in *H. pylori*-infected gastric mucosa, cathepsin X/Z (Ctsz) [10]. Its expression is mostly restricted to cells of the immune system, however the increase of Ctsz in gastric cancer was attributed to epithelial expression [11]. *H. pylori*-induced cytokine expression stimulates overexpression of Ctsz via ERK1/2 and JNK/p38 pathways in macrophages and epithelial cells, respectively [12]. Due to its unique carboxypeptidase specificity, Ctsz is unable to participate in bulk ECM degradation, thus questioning its direct contribution to the invasive processes of tumor cells [13]. The question of physiological or pathological functions for Ctsz is not yet fully clarified. Although reduced invasive capacity of tumor cells after Ctsz inhibition in Boyden chamber assays has been reported, the explanations for potential mechanisms are still questionable. Ctsz binds to integrins via its arginyl-glycyl-aspartic (RGD)-motif, thereby modulating adhesion and phagocytosis in macrophages, dendritic cell maturation, and T-cell migration [14], [15], [16], [17]. Recent findings indicate carboxyterminal processing of LFA-1 (integrin α_L_/β_2_) and bradikinin/kallidin, such that multifactorial interactions for Ctsz in immune response are proposed [18], [19].

Due to the complex interactions of cancer cells with inflammatory and other stromal cells during carcinogenesis, the afore mentioned *in vitro* studies, even when using primary gastric epithelial cells, were not able to fully clarify the *in vivo* functions of Ctsz in inflammation-driven carcinogenic processes. Here we present a gastritis mouse model with end points at 0, 24, 36, and 50 weeks post *H. pylori* infection to elucidate the effects of Ctsz deficiency for the relevant steps of early gastric carcinogenesis: acute gastritis, chronic inflammation, oxyntic atrophy, and finally metaplastic or early dysplastic lesions. In this model we examined the ability of *H. pylori* to colonize the wild-type and *ctsz^−/−^* stomachs, the differences in inflammatory response, the modulation of proinflammatory cytokines, as well as the development of epithelial lesions and cellular proliferation.

### Animals

Cathepsin X/Z-deficient mice (mutant allele *Ctsz^tm1Thre^;* here abbreviated *ctsz^−/−^*) were generated by gene targeting in mouse embryonic stem cells as previously described [20]. The mouse strain was backcrossed for 10 generations onto the C57BL6/N genetic background. The animals were housed in a specific pathogen-free (SPF) facility with 12∶12 light/dark cycle and unconditional access to water and food. In addition, stomachs from specific pathogen-free C57BL6/N (wt) and *ctsz* ^−/−^ mice were routinely analyzed to be negative for *H. pylori*. All experiments and procedures were conducted in accordance with the German National Guidelines for the Use of Experimental Animals (Animal Protection Act, Tierschutzgesetz, TierSchG), and were approved by the “Landesverwaltungsamt Sachsen-Anhalt” (AZ 42502-2-792 UniMD).

### Primary Gastric Epithelial Cells

For the isolation of primary gastric epithelial cells, uninfected wt and c*tsz^−/−^* mice were sacrified at 12 to 20 weeks, stomachs were removed and immediately cleaned in RPMI 1640 medium (Invitrogen, Karlsruhe, Germany). The stomachs were cut into small pieces (1–2 mm^2^) and incubated by constant stirring in 25 ml collagenase (Sigma)/dispase (Invitrogen) solution (12.000 U collagenase I, 125 U dispase, 125 mg BSA per 100 ml Quantum 286) for 2 hours at 37°C. Plates were precoated with Matrigel™ (5 µl/ml; BD Biosciences, Germany). Dispersed cells were washed and resuspended in epithelial cell culture medium Quantum 286 (PAA, Linz, Austria) supplemented with Gentamycin (10 µg/ml), Penecillin/Streptomycin (5 µg/ml, Invitrogen, Karlsruhe, Germany), and seeded onto Matrigel-coated plates (Biochrom, Berlin, Germany) [17].

### Cultivation of Helicobacter Pylori

The *H. pylori* strains SS1 (Sydney Strain 1) and B128, both mouse-adapted strains, were cultured in thin layers on 10% horse serum agar plates supplemented with vancomycin (10 µg/ml), trimethoprim (5 µg/ml), and nystatin (1 µg/ml), and incubated for 48 hours at 37°C in an anaerobic jar containing a campygen gas mix of 5% O_2,_ 10% CO_2,_ and 85% N_2_ (Oxoid, Wesel, Germany) as previously reported [21]. The *H. pylori* strain *SS1* was provided by Prof. Steffen Backert (School of Biomolecular & Biomedical Science, University College Dublin). For the infection, bacteria were harvested in BHI-medium.

### Experimental Infection

Animals were divided in four groups: (A) sham inoculated wt mice; (B) *H. pylori*-infected wt- mice; (C) sham inoculated *ctsz* ^−/−^ mice; and (D) *H. pylori*-infecetd wt- mice. At 8 weeks of age, wt and *ctsz* ^−/−^ mice of both genders were orally infected with 10^8^ cfu SS1 or B128 in 200 µl of BHI-medium or medium alone three times per week. For the infection of primary gastric epithelial cells, bacteria were harvested in PBS, pH 7.4, and added to the serum-starved cells at a multiplicity of infection (MOI) of 50∶1.

At 24, 36, and 50 weeks post infection (wpi), mice were euthanized by cervical dislocation. In each group at a specific time point, 10 mice were allotted for paraffin-embedding, and 5 mice for cryopreservation. The stomachs were removed and placed intact in 10% neutral buffered formalin for 24 hours. In our experience, performing longitudinal sections of the stomach into rings is the best way to present all parts of the stomach at once and to evaluate expansion of gastritis. For cryopreservation of tissue for RNA/DNA/protein extractions, the stomach was opened along the greater curvarture and extensively washed with sterile buffer. Tissue samples (20–50 mg) of antrum and corpus were snap frozen in liquid nitrogen [22].

### Histological Analysis

Deparaffinized serial sections stained with hematoxylin and eosin were graded for intensity of inflammation, glandular ectasia (cystically dialted glands), foveolar hyperplasia, and mucous metaplasia in a blinded manner. The presence of metaplasia was confirmed by periodic acid-Schiff/alcian blue stain. Scoring of infiltrating macrophages was done by labeling with the antibody F4/80 (AbDSerotech, Düsseldorf, Germany). For quantitative comparison of proliferation activity, we determined the labeling index of epithelial cells using anti-mouse Ki-67 antibody (Dako, Hamburg, Germany). Immunohistochemistry for Ctsz and cathepsin B (Ctsb) was performed using goat anti-mouse Cathepsin B antibody and goat anti-mouse Cathepsin X/Z antibody (R&D Systems, Wiesbaden, Germany), respectively. Scoring was done according to the criteria of Rogers et al. (2005) with ascending scales from 0 to 5 for gastric lesions [23]. Labeled nuclei and F4/80-positive cells in proximal corpus were counted per visual field at 200x magnification. Density and distribution of *H. pylori* were semiquantitatively assessed using Warthin-Starry staining (score 0–3). All histological and immunohistological sections were read in a blinded manner. A Nanozoomer Digital Pathology System (Hamamatsu, Herrsching, Germany) was used for archiving whole slide images at 0.23 µm/pixel resolution.

### Protein Extraction and Western Blotting

Frozen tissue samples and primary epithelial cells were homogenized in a phosphate buffer at pH 6.0 (50 mM sodium phosphate, 0.2 M NaCl, 5 mM EDTA, 100 µM E-64, 1 mM PMSF) by sonication. For the preparation of membrane and cytoplasmic fractions, a special protocol of Backert *et al.* was used [24]. Protein contents were measured in all samples using the Bio-Rad DC Protein Assay (Bio-Rad, Hercules, Ca). Fifty µg protein was separated by SDS-PAGE and blotted onto nitrocellulose membranes. The membranes were blocked with 3% dry milk in TBS/Tween and incubated for 2 hours at RT with goat anti-mouse cathepsin X/Z antibody (1∶500) and goat anti-mouse cathepsin B antibody (1∶500), which do not crossreact with other cathepsins (R&D Systems) as well as with mouse anti-CagA (1∶400, Aalto) and rabbit anti-mouse actin (1∶1000, Sigma). This is followed by incubation with the secondary, peroxidase-conjugated antibody (1∶25,000, R&D Systems) for 30 min. SuperSignal® chemiluminescence substrate (Milipore, Schwalbach, Germany) was used for detection. A MagicMark standard (Invitrogen, Karlsruhe, Germany) was used to identify the molecular weights. The ECL images were acquired and quantified using the GeneGnome and GeneTools image scanning and analysis package (Syngene BioImaging Systems, Synoptics Ltd.).

### Quantitative Real-time PCR Analysis

Assays were performed on the LightCycler™ (Roche Diagnostics, Mannheim, Germany). Several dilutions of plasmids containing cDNA fragments of different genes were used as internal controls. For plasmid construction, the cDNA fragments were amplified using the following primers: *H. pylori* (F, 5′ - CTG GAG AGA CTA AGC CCT CC –3′; R, 5′ - ATT ACT GAC GCT GAT TGT GC -3′) mIL-1β (F, 5′-CAACCAACAAGTGATATTCTCCATG-3′; R, 5′-GATCCACACTCTCCAGCTGCA-3′) mCxcl1/KC (F, 5′-GCACCCAAACCGAAGTCATAGC-3′; R, 5′-TTGTCAGAAGCCAGCGTTCACC-3′), mMCP-1 (F, 5′-GCTCTCTCTTCCTCCACCACCAT-3′; R, 5′-GCTCTCCAGCCTACTCATTGGGAT-3′), mIL-6 (F, 5-CACAAAGCCAGAGTCCTTCAGAGA-3′; R, 5-CTAGGTTTGCCGAGTAGATCTC-3′) and m-actin (F, 5-CTGGGTCATTCTTTTCACGGT-3′; R, 5-ACTGGGACGACATGGAGAAG-3′). The same primers were used for qPCR-reactions. PCR-products were inserted into the pCR2.1-TOPO vector (Invitrogen, Groningen, The Netherlands). The copy number of the resulting plasmids was calculated after DNA quantification.

Using TriFast™ reagent (Peqlab), we extracted total RNA from tissue samples according to the manufacturer‘s instructions. RNA was reverse-transcribed using Transcriptor High Fidelity cDNA Kit (Roche). PCR-reactions were performed using the qPCR Mastermix kit (Quantace, London) according to the manufacturer‘s instructions. The standard temperature profile included initial denaturation for 10 min at 95°C, followed by 35 cycles of denaturation at 95°C for 15 s, annealing at 52°C to 60°C (primer dependent) for 15 s, and extension at 72°C for 10 s. Mouse actin served as a housekeeping gene. The relative abundance (fold changes against uninfected controls) was calculated using the delta delta Ct method (2^−ΔΔCt^).

All oligonucleotide primers were purchased from BioTez (Berlin, Germany). The PCR-products were analyzed on a 1.8% agarose gel and visualized by ethidium bromide staining.

### Statistical Analysis

Data are expressed as Box plots or scatter plots (median values). GraphPad Prism software was used for statistical analysis. Statistical analyses were performed using the Mann-Whitney *U* test for unpaired groups. A p-value of 0.05 was set to be the level of significance. Systematic deviances between staining and quantitative PCR were tested using Bowker’s test. Furthermore, the level of agreement was evaluated using Cohen’s kappa. All tests were deliberately carried out to the full level of significance. Analyses were made using SPSS Statistics 19.0 (IBM Corporation, NY, USA).

## Results

**Figure 1:**
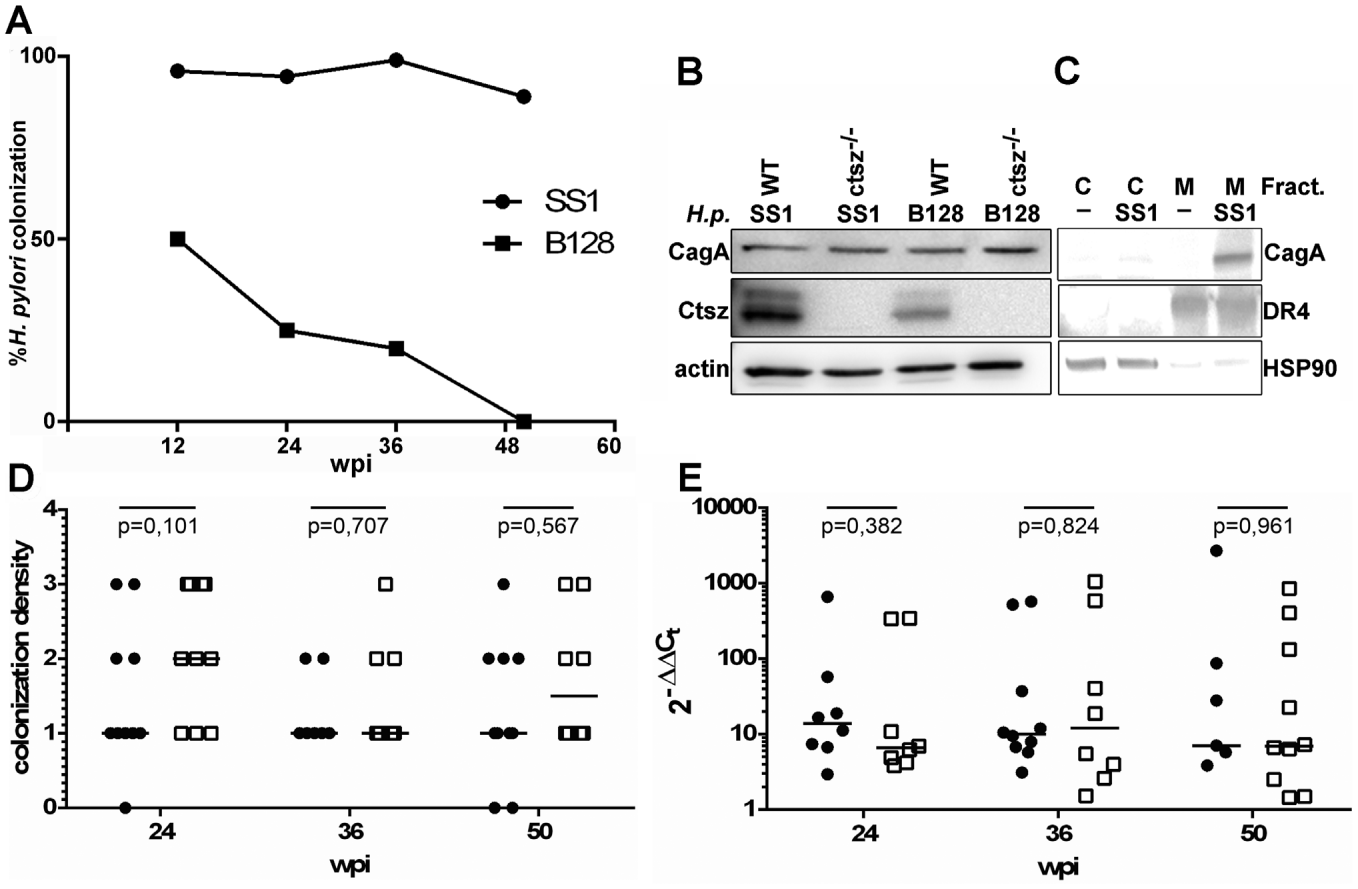
Colonization efficiency of *H.* *pylori* in the corpus mucosa and the induction of Ctsz. (A) Warthin starry staining revealed a stable colonization of the SS1 strain over 50 wpi, whereas the B128 strain failed to stably colonize the mouse mucosa for more than 24 weeks. Results are shown as % of *H. pylori*-positive tested animals for 12, 24, 36, and 50 wpi with animal numbers of 15/20, 16/20, 11/14, and 7/18 (B128/SS1), respectively. (B) Western blots of cell lysates showed induction of Ctsz in *H. pylori* SS1-, as well as B128-inoculated wt epithelial cells whereas *ctsz^−/−^* cells remained unaffected. CagA was detected in all infection experiments (B) without showing delivery into the cytoplasm in fractionated cells (C). Colonization density of corpus mucosa in C57BL/6 wt mice (•) and *ctsz^−/−^* (□) challenged with *H. pylori* SS1 for 24, 36 or 50 weeks was semiquantitatively graded of *H. pylori* levels using Warthin-Starry staining with scores from minimum = 1 to maximum = 3 (D) and quantified using the ΔΔCt method by qRT-PCR (E).

Wt and c*tsz* ^−/−^ mice were inoculated with either *H. pylori* SS1 or B128 strain (n = 49). According to the results of quantitative *H. pylori*-PCR and histological tests, at 12 weeks, almost all SS1-inoculated (96%), but only 50% of B128-inoculated mice were infected. 36 wpi 80% of B128-inoculated mice were *H. pylori*-negative, and 50 wpi no B128-infection was detected in either wt or c*tsz* ^−/−^ mice (Figure 1A).

As the *H. pylori* strain SS1 is known to efficiently colonize the gastric mucosa of mice despite a non-functional type IV secretion system (T4SS), we first had to determine whether this strain would be able to induce Ctsz upregulation in mice. Primary gastric epithelial cells of wt and c*tsz* ^−/−^ mice were infected with SS1 and B128 for 8 hours. Western blot analyses revealed a strong upregulation of Ctsz in both SS1- and B128-infected wt cells, which have no detectable Ctsz expression in the uninfected state. Surprisingly, all infected cells were screened and found to be positive for CagA (Figure 1B). Cellular fractionation of SS1-infected wt cells indicated that CagA was attached to the cell membranes and was not detected in cytoplasm (Figure 1C).

Hence, wt and c*tsz* ^−/−^ mice were infected with *H. pylori* SS1 and the colonization density was controlled in 1 animal per infection group at 12 wpi. Only infection groups with positive results were further challenged for 24 wpi, 36 wpi, and 50 wpi. Six to ten mice per group were sacrificed, the stomachs removed, fixed, and paraffin-embedded. To determine if potential differences in gastritis development were due to altered *H. pylori* colonization density in wt and c*tsz* ^−/−^ mice, Warthin-Starry staining (Figure 1D) and quantitative RT-PCR (Figure 1E) were performed to determine the *H. pylori* burden. *H. pylori* colonization was found to be stable over the time course of the experiment in both strains of mice. No significant systematic deviances between *H. pylori* staining and categorization of quantitative PCR were found (p = 0.371), although yielding a small level of agreement (kappa = 0.347) (Figure S1). Furthermore, there were no significant differences in *H. pylori* colonization intensity between infected wt and *ctsz* ^−/−^ mice over the time of 50 wpi. Sham incolutated mice were negative for *H. pylori* infection.

**Figure 2:**
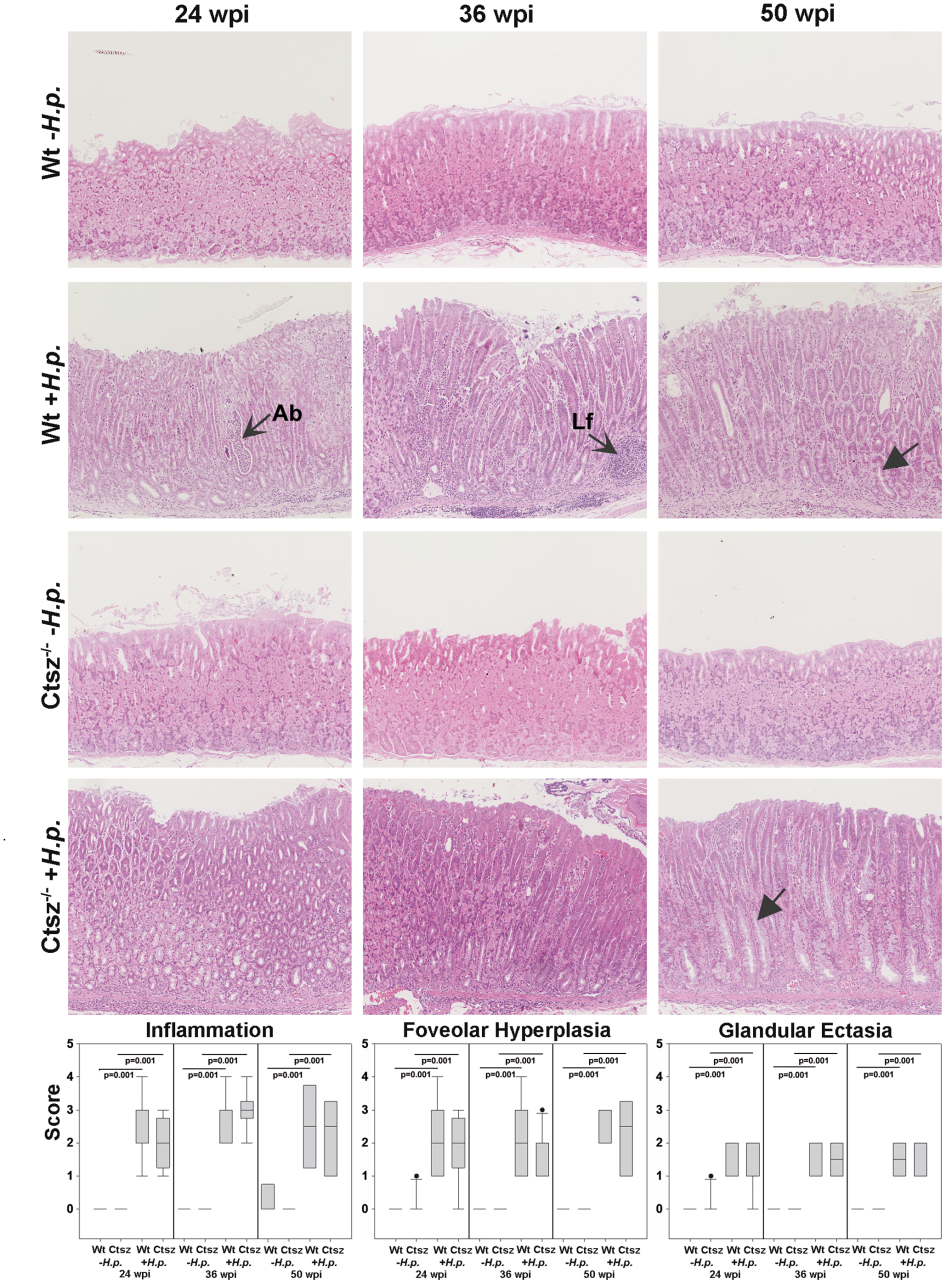
Histological evaluation of inflammation, hyperplasia, and glandular ectasia. Blinded H&E-stained gastric sections from n = 5–11 wt and *ctsz^−/−^* mice infected or non-infected with *H. pylori* SS1 for 24, 36, or 50 weeks were assessed. Sections were graded from 0–5 based on the criteria of Rogers *et al*. [23]. Compared to sham-inoculated mice, gastric mucosa of infected mice exhibited marked inflammation (*p* = 0.001) with abscesses (Ab) and lymph follicles (Lf), as well as mucosal thickening (*p* = 0.001), glandular ectasia (*p* = 0.001), and loss of parietal cells with development of mucus metaplasia (closed arrows). There were no statistically significant differences between wt and *ctsz^−/−^* mice for all three criteria. All box plots show 25^th^ to 75^th^ percentiles (box) and 5^th^ to 95^th^ percentiles (whiskers). Solid dots are outliers above 95%. The line in the box represents the median.

Paraffin sections (3 µm) stained with hematoxylin & eosin were assessed for morphological changes by *H. pylori* infection at 24, 36, and 50 wpi. In particular inflammation, epithelial cysts, foveolar hyperplasia, and metaplasia were evaluated in detail using a paradigm according to Rogers et al., with scores from 0 to 5 [23]. There was no evidence of gastric inflammation in uninfected control mice of wt and *ctsz* ^−/−^ origin until 50 wpi (Figure 2, wt and *ctsz* ^−/−^ -*H.p.*). Independent of Ctsz expression, all *H. pylori*-infected mice showed statistically significant infiltration of inflammatory cells between 24 and 50 wpi (Figure 2, wt and *ctsz* ^−/−^ +*H.p.*, *p* = 0.001). Abscesses and lymph follicles (open arrows) were frequently seen in both mice strains without detectable disparities. Similar results were obtained by analyzing the development of foveolar hyperplasia and formation of glandular ectasia or cysts. No significant differences were found between mouse strains or time points (Figure 2, wt and *ctsz* ^−/−^ +*H.p.*), and all the gastritis-associated lesions were found predominantly in the cardia and proximal corpus.

**Figure 3:**
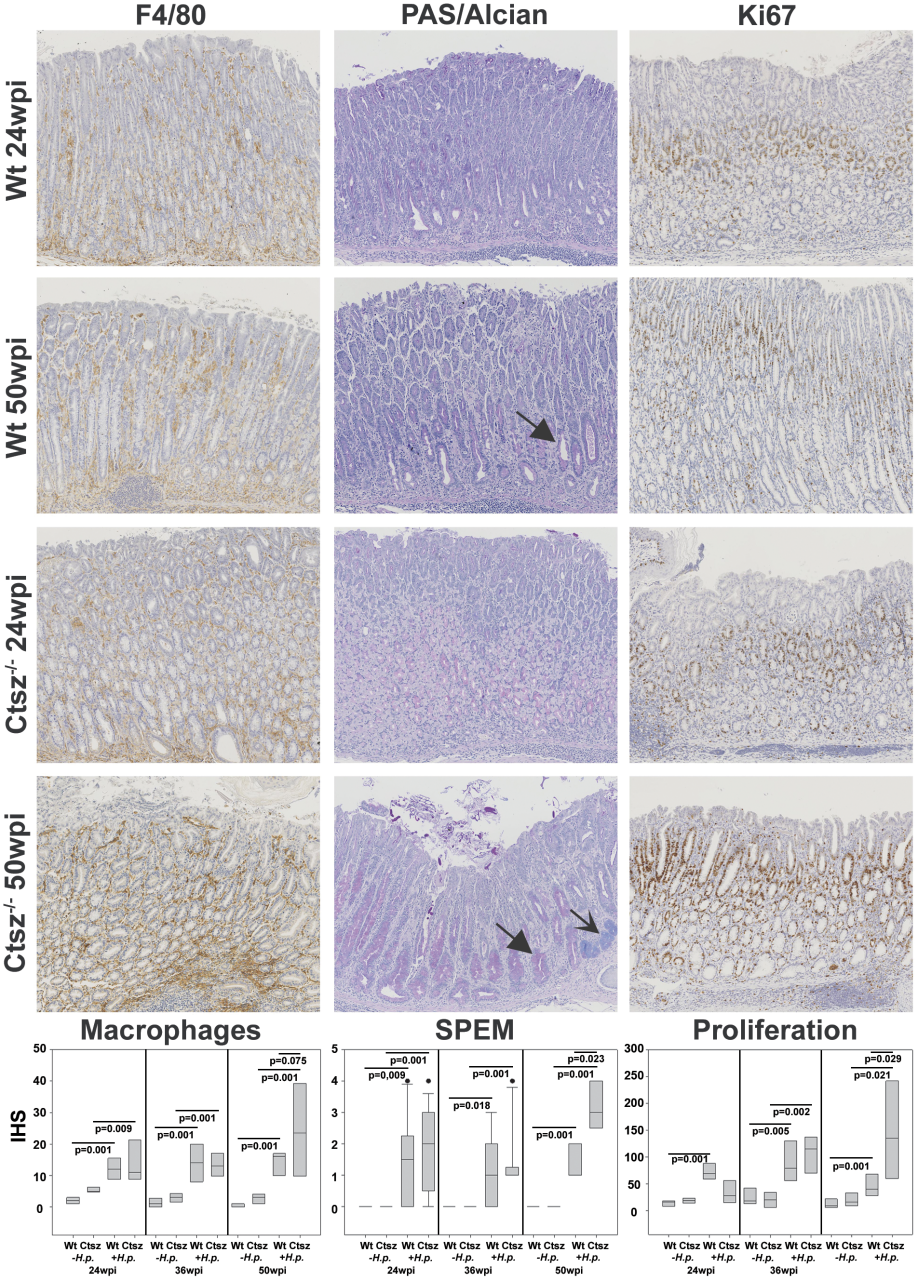
Histochemical (PAS/Alcian blue) and immunohistochemical (F4/80, Ki-67) stainings in gastric mucosa. Uninfected and *H. pylori* SS1-infected mice at 24 and 50 wpi were analyzed for proliferative activity, macrophage infiltration, and SPEM development. Expression of F4/80, indicating infiltrating macrophages, was much higher (*p* = 0.075) in infected *ctsz^−/−^* mice compared to wt at 50 wpi. This was accompanied by a higher proliferation rate as shown by nuclear Ki-67 immunoreactivity (p = 0.029) and significantly stronger SPEM formation (p = 0.023) in *ctsz^−/−^* mice (closed arrows) with intestinal-type acidic mucin-expressing glands (open arrows). Macrophages and proliferating cells were evaluated for their quantity per visual field. SPEM was quantified as outlined by Rogers et al. [23]. Results from data sets (n = 5–11) are presented in the box plots (IRS, immunoreactive score). All box plots show 25^th^ to 75^th^ percentiles (box) and 5^th^ to 95^th^ percentiles (whiskers). Solid dots are outliers above 95%. The line in the box represents the median.

As we have already described the importance of infiltrating Ctsz-positive macrophages in mediating several signaling pathways in *H. pylori* infection, we scored infiltrating F4/80-positive cells in infected versus non-infected wt and *ctsz* ^−/−^ mice [12], [17]. There were only a few F4/80-positive cells found in normal gastric mucosa in both *ctsz* ^−/−^ and wt mice. 24 wpi with *H. pylori*, immunohistochemistry revealed a significant increase of infiltrating F4/80-positive cells in gastric mucosa of *ctsz* ^−/−^ (p = 0.009) and wt mice (p = 0.001). Compared to wt animals with no further increase in F4/80 positivity, *ctsz* ^−/−^ mice exhibited a prominent infiltration (p = 0.075) of F4/80-positive cells after 50 wpi (Figure 3, left). This was associated with a higher epithelial proliferation index (*p* = 0,029) at 50 wpi only in infected *ctsz* ^−/−^, but not in wt mice as assessed by morphometric analysis of Ki-67 staining (Figure 3, right). Typically, Ki67-positive cells present as a small band within the isthmic regions of gastric glands in uninfected wt and *ctsz* ^−/−^ mice. After infection, the proliferative compartment was expanded with high scores of Ki67 in the bottom of hyperplastic glands and at sites of regeneration.

Spasmolytic polypeptide-expressing metaplasia (SPEM) is associated with progression to gastric cancer [1]. Interestingly, *ctsz* ^−/−^ mice also exhibited a significantly more severe metaplasia (*p* = 0.023, closed arrows) associated with oxynthic atrophy at 50 wpi compared to wt animals (Figure 3, middle). Intestinal-type acidic mucin-expressing glands were predominantly detected in *ctsz^−/−^* mice (Figure 3, open arrow).

**Figure 4:**
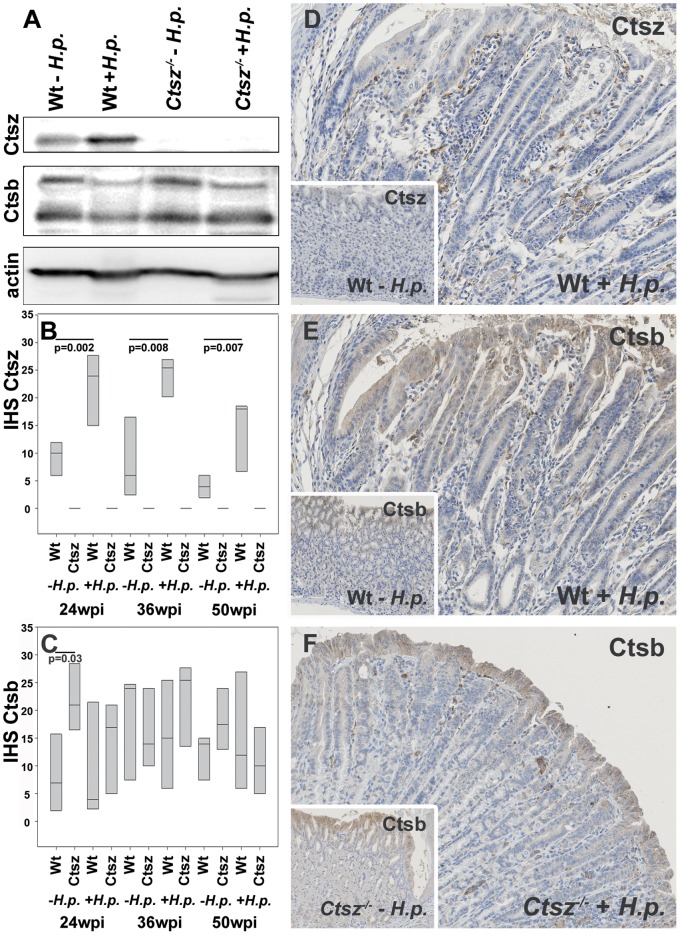
Differential expression of Ctsz and Ctsb. (A) Western blot analysis revealed *H. pylori*-dependent induction of Ctsz expression in wt mice after 36 wpi, whereas Ctsb remains unaffected in both *ctsz^−/−^* and wt mice. (B and C) Immunohistochemical (IHC) grading and (D–E) staining of Ctsz and Ctsb of corpus mucosa from wt (D,E) and *ctsz^−/−^* mice infected and uninfected (inserts) with *H. pylori* SS1. *H. pylori* infection significantly increased Ctsz expression in macrophages and deep glands, but only slightly in surface epithelium (*p* = 0.002–0.008). Ctsb was predominantly expressed in surface epithelium and some macrophages with no significant changes after *H. pylori* infection. All box plots show 25^th^ to 75^th^ percentiles. The line in the box represents the median.

Earlier reports have described a compensatory effect of Ctsz in *ctsb* ^−/−^ mice, as well as a trend towards higher Ctsb mRNA expression in *ctsz* ^−/−^ mice [17], [20]. In the present study, we analyzed Ctsz and Ctsb protein expression in tissue lysates and spatial distribution in paraffin sections of proximal corpus comparing wt and *ctsz* ^−/−^ mice. Ctsz expression was absent in *ctsz* ^−/−^ mice, but increased significantly (p≤0.002), depending on *H. pylori* infection after 24 wpi in wt mice (Figure 4A,B,D). Surprisingly, Ctsb was only minimally increased in non-infected *ctsz* ^−/−^ mice (Figure 4A,C,F). In line with published human data, Ctsb expression was not induced by *H. pylori* in either wt or *ctsz* ^−/−^ mice (Fig.4C,E,F).

If expressed, both enzymes show a cytoplasmic staining pattern. Ctsz was predominantly expressed in macrophages, infected surface epithelium, and gastric glands, Ctsb in the surface epithelium and inflammatory cells. Ctsz seems to switch from an apical expression in surface epithelium and foveolae to a basal expression most notably in deep gastric glands. SPEM is generally negative for Ctsz.

**Figure 5:**
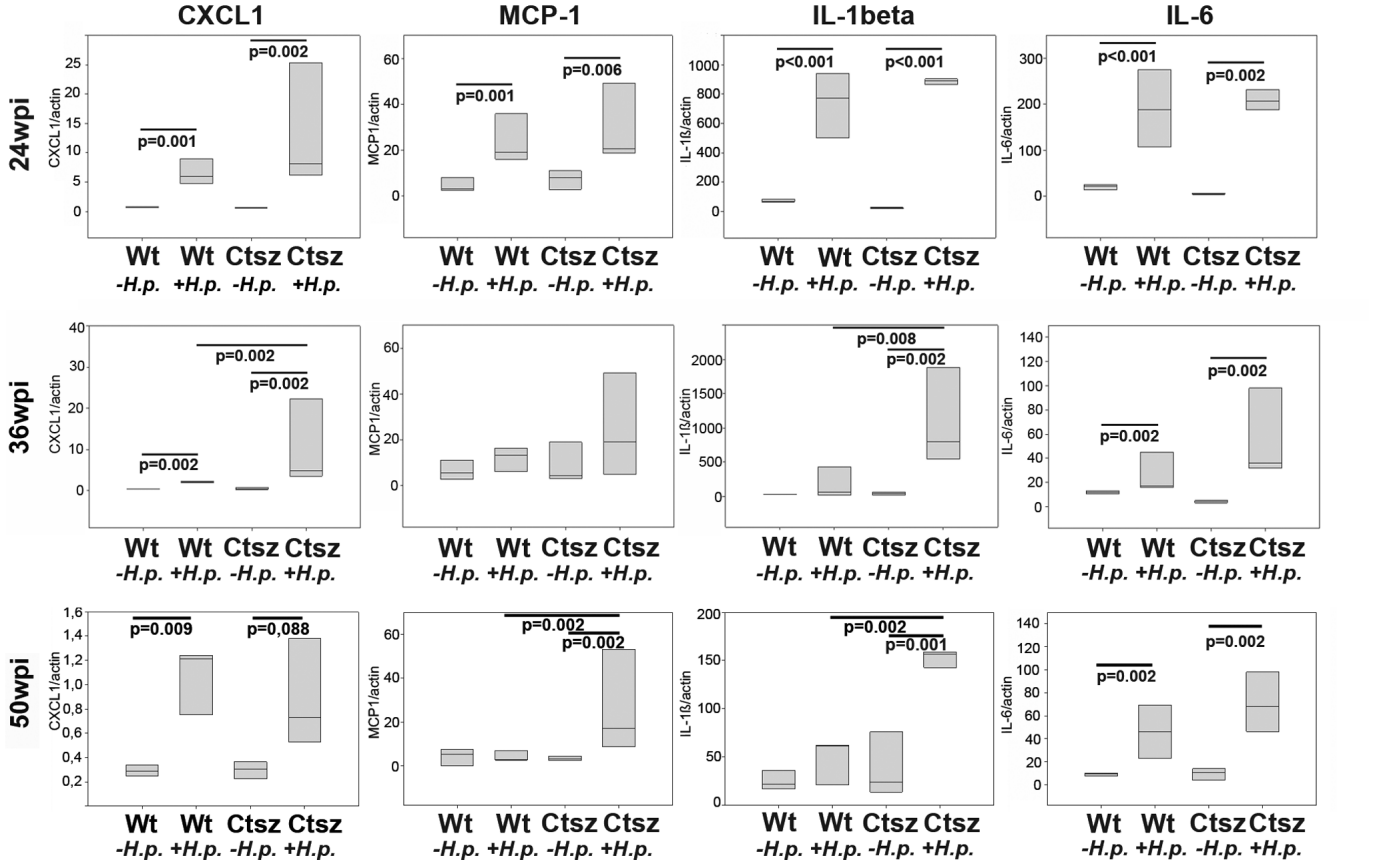
Proinflammatory cytokine response. Quantitative RT-PCR for CXCL1, MCP-1, IL-1β, and IL-6 in the infected versus uninfected wt and *ctsz^−/−^* stomachs at 24, 36, and 50 wpi. All box plots show 25^th^ to 75^th^ percentiles. The line in the box represents the median.

Compared to wt animals after long term *H. pylori* infection, *ctsz* ^−/−^ mice showed a significantly increased number of macrophages, suggesting that Ctsz deficiency may trigger the immune responses to chronic inflammation. Reverse transcription-PCR (RT-PCR) on samples of *H. pylori*-infected or BHI-treated mice revealed an increase of CXCL1, MCP-1, IL-1β, and IL-6 after 24 weeks of *H. pylori* infection in each mouse strain. CXCL1 and MCP-1 tend to be more frequently induced in *ctsz* ^−/−^ mice than in wt mice. More interestingly, while there was no induction of cytokines in wt mice at 36 wpi, the upregulation in *ctsz* ^−/−^ mice is mostly stable up to 36 wpi (Figure 5).

## Discussion

Several animal models of *H. pylori* infection have been described, ranging from nonhuman primates to mice. Since it is difficult to keep larger organisms under experimental conditions, Mongolian gerbils and mice are now generally accepted as model systems. Although Mongolian gerbils closely mimic human disease, this model is to a large extent limited by the paucity of reagents and knockout variants [25]. Mice have been successfully infected with several strains of *H. pylori.* These are mostly CagA^−^ or *cag*-PAI-defective, including the mouse-adapted Sydney strain-1 (SS1). Infection of mice with *H. pylori cag^+^* strains frequently leads to deletions within the *cag-*PAI and to reduced ability of CagA translocation of re-isolates after 4–12 weeks of infection [26], [27]. The selection of an appropriate *H. pylori* strain is therefore a difficult, but critical step in the experimental design. Our aim was to investigate the influence of Ctsz-deficiency on the *H. pylori*-dependent etiopathology in long-term experiments (several months) until preneoplastic lesions develop. In our investigations, inoculation with the cagA^+^ strain B128 resulted in a rapid upregulation of Ctsz. Indeed, infection was not persistent longer than 24 weeks without further progression of gastritis. The SS1 strain was also able to induce Ctsz overexpression in gastric epithelial and stromal cells, and showed stable colonization rates over 50 wpi with development of moderate to severe gastritis and SPEM starting at 24 wpi. Obviously, there might be a total increase in Ctsz expression in mouse stomachs in a CagA-independent manner. Perhaps the SS1-induced increase of proinflammatory cytokines, shown by us previously *in vitro* and here *in vivo,* is responsible for the Ctsz upregulation in epithelium and macrophages [17]. The *H. pylori* SS1 infection is the most commonly used and the most standardized mouse model at present, and comprises all criteria needed for our experiments. However, care should be taken regarding the interpretation and discussion of the results in the context of contributions of various *cag*PAI genes.

Based on the findings that Ctsz has immunomodulatory properties and that it is overexpressed in *H. pylori*-infected gastric mucosa and cancer, we used Ctsz-deficient mice to compile specific functions of this cysteine protease in the process of inflammation and premalignant epithelial changes associated with chronic *H. pylori* gastritis. The first key point was the analysis of the adhesion properties and persistence of *H. pylori* in *ctsz* ^−/−^ compared to wt mice. *H. pylori* is known to have strong binding affinity for heparin sulphate proteoglycan, and CagL was suggested to bind via its RGD motif to integrins α_5_ß_1_ and α_V_ß_3_ [28], [29], [30]. Ctsz happened to have the same binding properties. Heparin-Ctsz interaction is specific and promotes an increase in catalytic activity. Heparan sulphate proteoglycans are related to insertion processes of Ctsz at cell surface controlling cellular adhesion processes [15]. Lechner et al. provided evidence that pro-Ctsz is capable of interacting with integrin α_V_ß_3_ through an RGD-dependent mechanism, resulting in altered adhesive properties [14]. As we did not find any significant differences in quantity or localization of *H. pylori,* we do not think that Ctsz and *H. pylori* compete for binding sites or interact to constrain binding of *H. pylori*. It is questionable if the human processes outlined above can be transferred to the mouse. At this point, no conclusion can be drawn regarding the interaction of Ctsz and *H. pylori* at sites of integrin binding. This needs to be further analyzed in primary human cell cultures.

Since we found the upregulation of Ctsz to correlate with the severity of gastric injury, starting with mild gastritis up to intestinal cancer, a significant decrease of inflammation and epithelial defects was postulated for *H. pylori*-infected *ctsz* ^−/−^ cells [11], [12]. As the contrary is true, the following question arises: which is the mechanism behind the ability of *Ctsz*-deficiency to upregulate macrophage infiltration and metaplastic transition. Similarly unexpected findings were published for MMP-7. The authors suggested that knockout of MMP-7 should decrease the inflammatory response to *H. pylori* but, in fact, MMP-7 deficiency enhanced the Th-1 and Th17-mediated responses after *H. pylori* SS1 infection. They postulated an inability of *mmp-7^−/−^* mice to establish proper chemoattractant gradients, thus preventing transepithelial migration of immune cells, manifesting in increased inflammation [7]. Such an explanation could also apply to Ctsz with the restriction that macrophages, not B- or T-cells, infiltrated knockout mice more strongly (data not shown). The induction of different cytokines in *ctsz* ^−/−^ mice tends to be stronger and remains stable over a long period of time, although in wt mice, only insufficient induction was seen at 36 and 50 wpi. CXCL1/KC, MCP-1, and IL-6 are known to strengthen neutrophil chemoattractant activity, recruitment of monocytes, memory T cells, and differentiation of B-cells, respectively. Their long-lasting increase could explain the scores of infiltrating macrophages at 50 wpi in *ctsz* ^−/−^ mice. Anyway, levels of lymphocytes and granulocytes are indistinguishable in infected wt and *ctsz* ^−/−^ stomachs (data not shown). This was the second unexpected finding because Ctsz has been described to promote T-cell migration by cleaving the β_2_ cytoplasmic tail of LFA-1 (lymphocyte function-associated antigen) [19]. In this context, it is questionable if in rodents the functions of LFA-1 are similar to those in humans. CD11a-deficient mice showed normal responses to systemic infections [31]. Furthermore, in contrast to human CD4^+^ cells, primary murine CD4^+^ T cells were resistant to treatment with *H. pylori* harboring the vacA gene with an m1 allele. This effect could be abrogated by expression of human LFA-1 in the murine Tcells [32].

A recent study has demonstrated a causal relationship between epithelial polarity and proliferation control. In polarized epithelial cells, CagA-driven ERK signals prevent p21^Waf1/Cip1^ expression and induced mitogenesis. In nonpolarized epithelial cells, ERK activation results in oncogenic stress, up-regulation of p21^Waf1/Cip1^ cyclin-dependent kinase inhibitor, and induction of senescence [33]. Accelerated cellular senescence has also been described in Ctsz-deficient murine embryonic fibroblasts or siRNA-transfected human dermal fibroblasts accompanied by increased expression levels of p21 [34]. These findings are in line with ours because cellular senescence is defined by cell cycle arrest and suppresses cellular proliferation. Although we found overall significant increased Ki67 expression in long-term *H. pylori*-infected *ctsz* ^−/−^ stomachs, we detected significantly more SPEM in *ctsz* ^−/−^ mice, and these metaplastic cells were Ki67-negative. Of course, SPEM does not arise from epithelial-mesenchymal transition, but execution of the cell differentiation program requires G1 cell-cycle arrest. Here, CagA causes G1-arrest by inducing p21 and deregulates the β-catenin signal. Ectopic co-expression of p21 and constitutively active β-catenin resulted in an induction of MUC2, which has been reported to be involved in intestinal metaplasia [35]. Furthermore, PymT^+/−^;*ctsz* ^−/−^ mice showed reduced cell death in mammary tumors, resulting in enlarged tumors compared to wt or Ctsb^−/−^ variants [20]. Altogether, Ctsz-deficiency could be able to boost or even to substitute *H. pylori*-dependent pathways, resulting in epithelial differentiation.

Our data show an active role for Ctsz in chronic inflammation and the development of gastric metaplasia. Ctsz is involved in the regulation of cytokine expression and thereby in transepithelial macrophage migration. Whether or not a high number of infiltrating macrophages are protective or risk factors for etiopathology needs to be elucidated in a recently established corresponding gastric cancer model (Krueger *et al*., manuscript in preparation). Hopefully, the results from these *ctsz* ^−/−^;INSGAS mice will support our hypothesis for a protective role of Ctsz in metaplastic differentiation.

## Supporting Information

Figure S1 **Colonization density of corpus mucosa in C57BL/6 wt and** ***ctsz^−/−^*** **mice challenged with** ***H. pylori*** **SS1 for 24, 36 or 50 weeks was semiquantitatively graded of** ***H. pylori*** **levels using Warthin-Starry staining with scores from minimum = 1 to maximum = 3 and quantified using the ΔΔCt method by qRT-PCR.** Systematic deviances between staining and quantitative PCR were tested using Bowker’s test, the level of agreement was evaluated using Cohen’s kappa.
(TIF)
