# Supplementary figures and images for: Induction of Premalignant Host Responses by Cathepsin X/Z-Deficiency in Helicobacter Pylori-Infected Mice
Source: PLoS One. 2013 Jul 30;8(7):e70242. doi: 10.1371/journal.pone.0070242 (PMC3728094; doi:10.1371/journal.pone.0070242)

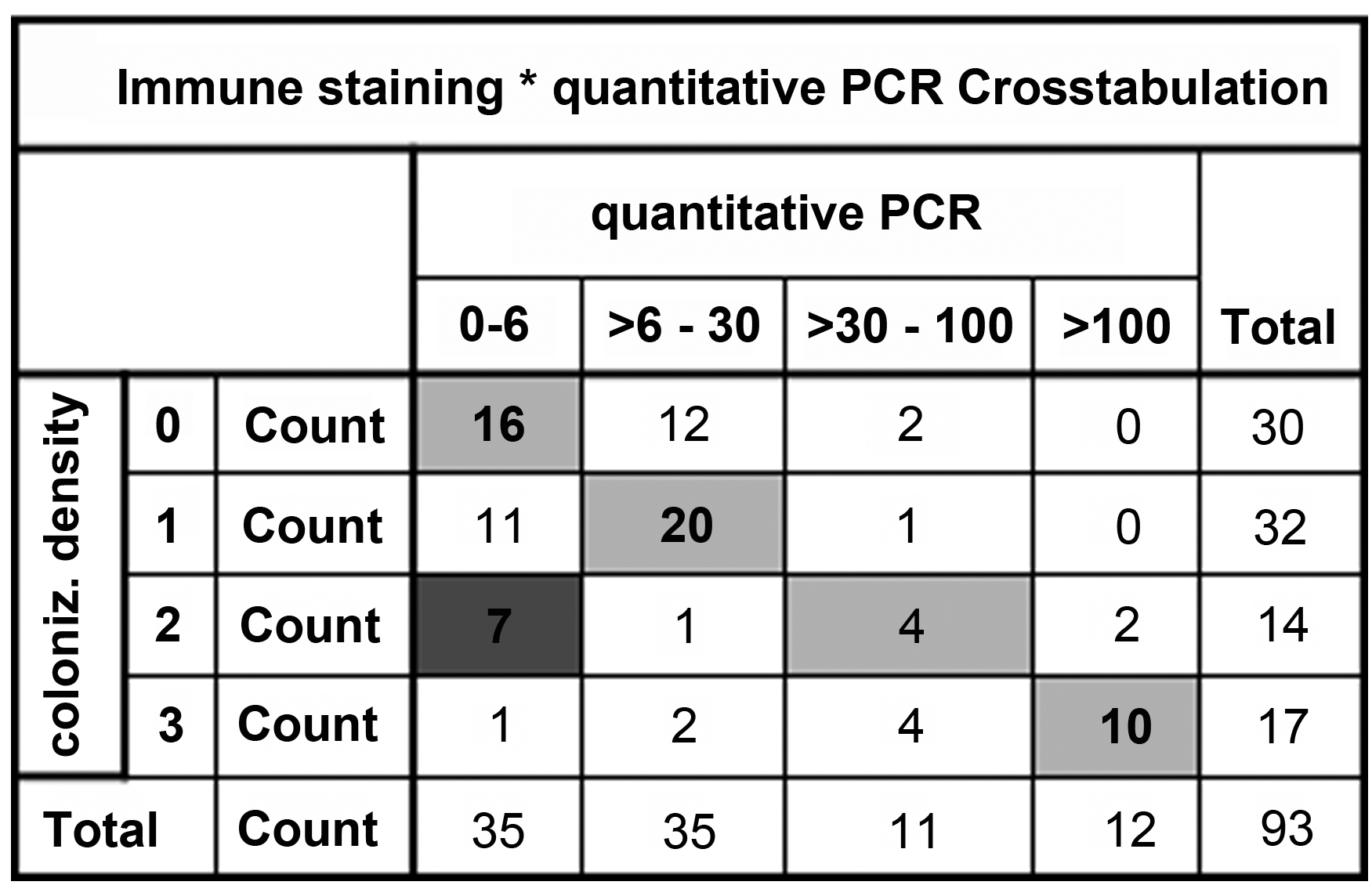

Supplement: Figure S1 — Colonization density of corpus mucosa in C57BL/6 wt and ctsz−/− mice challenged with H. pylori SS1 for 24, 36 or 50 weeks was semiquantitatively graded of H. pylori levels using Warthin-Starry staining with scores from minimum = 1 to maximum = 3 and quantified using the ΔΔCt method by qRT-PCR. Systematic deviances between staining and quantitative PCR were tested using Bowker’s test, the level of agreement was evaluated using Cohen’s kappa. (TIF) [file pone.0070242.s001.tif]
